# Gender differences of polymorphisms in the TF and TFPI genes, as related to phenotypes in patients with coronary heart disease and type-2 diabetes

**DOI:** 10.1186/1477-9560-8-7

**Published:** 2010-05-05

**Authors:** Trine B Opstad, Alf Åge Pettersen, Thomas Weiss, Harald Arnesen, Ingebjørg Seljeflot

**Affiliations:** 1 Center for Clinical Heart Research, Department of Cardiology, Oslo University Hospital Ulleval, Oslo, Norway; 2 Center for Clinical Research, Oslo University Hospital Ulleval, Oslo, Norway; 3 Faculty of Medicine, University of Oslo, Norway

## Abstract

**Background:**

Tissue factor (TF) and its inhibitor tissue factor pathway inhibitor (TFPI) are the main regulators of the initiation of the coagulation process, important in atherothrombosis. In this study we have investigated the frequency of six known TF and TFPI single nucleotide polymorphisms (SNPs) in CHD patients as compared to healthy individuals. These genotypes and the phenotypes (TF, TFPI free and total antigen) were evaluated with special reference to gender and diabetes in the CHD population.

**Methods:**

Patients with angiographically verified CHD (n = 1001; 22% women, 20% diabetics), and 204 healthy controls (28% women), were included. The investigated SNPs were: TF -1812C/T and TF -603A/G in the 5'upstream region, TF 5466A/G in intron 2, TFPI -399C/T and TFPI -287T/C in the 5'upstream region and the TFPI -33T/C in intron 7.

**Results:**

No significant differences in frequencies between the CHD population and the controls of any polymorphisms were observed. In the CHD population, the TF 5466 A/G SNP were significantly more frequent in women as compared to men (p < 0.001). The TF-1812C/T and the TF-603A/G SNPs were significantly more frequent in women without type-2 diabetes compared to those with diabetes (p < 0.018, both), and the heterozygous genotypes were associated with significantly lower TF plasma levels compared to the homozygous genotypes (p < 0.02, both).

The TFPI-399C/T and the TFPI-33T/C SNPs were associated with lower and higher TFPI total antigen levels, respectively (p < 0.001, both).

**Conclusion:**

Genetic variations in the TF and TFPI genes seem to be associated with gender and type-2 diabetes, partly affecting their respective phenotypes.

## Background

Tissue factor (TF) and its endogenous inhibitor, tissue factor pathway inhibitor (TFPI) are the main regulators of the initiation of the coagulation process, important in atherothrombosis. Injury of the vessel wall and rupture of an atherosclerotic plaque lead to exposure of TF to circulating blood, followed by an activation of the haemostatic system. In addition, blood-borne, or soluble TF (TF) from micro particles and monocytes may represent thrombogenic potential [1]. TFPI is the main regulator in the initial step of the coagulation cascade mediated by TF, by binding to coagulation factors Xa, VIIa and TF forming an inactive complex [2,3]. TFPI, mainly produced by the endothelium [4], is predominantly associated with lipoproteins in the blood [2,5] or is endothelial-bound [6], whereas a small portion circulates as free molecules. Enhanced TF expression has been found in atherosclerotic plaques [7], in which TFPI has been found to co-localize [8].

Elevated levels of TF has been associated to cardiovascular disease (CVD) [9-11] and also shown to be predictive for future events in some studies [12,13], whereas the role of TFPI in atherothrombosis remains unclear. However, a positive correlation between plasma levels of TF and TFPI in ischemic heart disease has been demonstrated [9].

The association between CVD and type-2 diabetes mellitus (T2DM) is well established. It has also been shown, that women with coronary heart disease (CHD) more often present T2DM than men [14,15], however, the cause and molecular mechanism underlying this relationship is not fully explored. The atherosclerotic process includes both environmental and multiple genetic factors, leading to the unfavorable state in the cardiovascular system. The accelerated atherosclerosis seen in diabetic patients might be a consequence of chronic hyperglycemia [16]. Permanent hyperglycemia may lead to glycosylation of proteins, including albumin, and the latter has been shown to increase the expression of TF in monocytes [17]. Further, elevated levels of circulating TF-procoagulant activity have been demonstrated in patients with T2DM [18]. Hyperglycemia may also lead to dysfunctional endothelium in which TFPI, suggested to be a marker of endothelial dysfunction, may be affected [19]. Both genetic and environmental risk factors are able to modify the expression of proteins, and several genetic variants in the TF and TFPI gene have been investigated in relation to CHD [20-26]. Single nucleotide polymorphisms (SNPs) may affect the levels of their gene encoding products, leading to a modified susceptibility to CHD.

In the present study we aimed to investigate the frequency of six known SNPs in the genes coding for TF and TFPI in patients with CHD, as compared to healthy individuals, and furthermore to explore the influence of these SNPs on plasma levels of the proteins, with special emphasis on diabetes and gender differences in a CHD population. The choice of the investigated polymorphisms is based on previous research in the field.

The results of the present study indicate differences in frequencies of the TF polymorphisms as related to diabetes and gender, and additionally changes in plasma levels according to the different TF and TFPI SNPs.

## Methods

### Study population

We studied 1001 patients enrolled in the Aspirin non-responsiveness and clopidogrel clinical endpoint trial (ASCET) [27]. All patients had angiographically verified stable CHD and 97% were Caucasians. The mean age was 62 years and 22% were women. Twenty percent had T2DM, defined as known T2DM or fasting glucose > 7 mmol/l, 44% had experienced an acute myocardial infarction (MI). The control group consisted of 204 apparently healthy controls, mean age 55 years, 28% being women, all Caucasians. They were included after clinical examination and an ECG-test, to rule out any clinical evidence for atherosclerotic disease. The study was approved by the Regional Ethics Committee and all patients gave their written informed consent to participate. The ASCET study is registered at the website; clinicaltrials.gov, with the identification number: NCT00222261

### Blood sampling

Venous blood was collected by standard venipuncture between 8 and 10 a.m. in fasting condition and without intake of any medication. Citrated plasma (0.129 M in dilution 1:10), stored on ice, was separated within 30 minutes by centrifugation at 4°C and 3.000 × g for 20 minutes. For genotype determination, also performed in the healthy controls, whole blood collected in EDTA tubes was used. All samples were stored at -80°C until analyzed. Serum lipids, glucose and HbA1c, were determined by conventional routine methods.

### DNA isolation

DNA was purified from whole blood (EDTA) on the Magna Pure LC Instrument (Roche Diagnostics GmbH, Mannheim, Germany), using MagNA Pure DNA LC isolation kit, Large Volume (Roche Diagnostics GmbH). The DNA Large Volume Blood Protocol of the MagNA Pure software program, version 3.0, was used for extraction with settings for an initial sample volume of 500 μl and an elution volume of 100 μl. DNA purity and quantity were tested on the NanoDrop, ND-1000 (Saveen Werner, Sweden) and DNA was kept at -80°C until analyzed.

### Genotype analysis

The TF -1812C/T (rs 958587) and TF -603A/G (rs 1361600) in the 5'upstream region, TF 5466A/G (rs3917643) in intron 2, the TFPI -399C/T (rs 10153820) and TFPI -287T/C (rs 10931292) in the 5'upstream region, and the TFPI -33T/C (rs 8176592) in intron 7 polymorphisms were investigated. Further, the haplotype TF I (TF -1812CC combined with TF 5466 AA/AG) was investigated. Allelic discrimination was performed by the Applied Biosystems 7900HT Fast Real-Time PCR system, using allele specific primers and probes included in the Taqman SNP Genotyping assay (TaqMan MGB probes; FAM and VIC dye-labeled) and the TaqMan Genotyping Master Mix, (Applied Biosystems, Foster City, CA, USA). A final reaction volume of 25 μl was used and 1-20 ng of genomic DNA was added. The Thermal Cycler Conditions were the same for all polymorphisms with an initial step of 10 min at 95°C followed by 47 cycles of 15 sec at 92°C and 1 min at 60°C each.

### Plasma TF and TFPI analyses

Levels of TF and TFPI were measured using commercial ELISA kits; the Imubind TF kit, recognising TF-apo, TF and TF-VII complexes (American Diagnostic Inc., Greenwich, CT, USA), and the Asserachrom TFPI free antigen and Asserachrom TFPI total antigen kits, recognising the full-length TFPI molecules, and the full-length and truncated TFPI molecules including TFPI bound to lipoproteins, respectively (Stago Diagnostica, Asnière, France). The inter-assay coefficients of variation in our laboratory were 7.9%, 5.6% and 3.8%, respectively.

### Statistical analysis

The *x*^2 ^test was used to test for deviation of the genotype distribution from Hardy-Weinberg equilibrium. Allele frequencies were evaluated by gene counting and group differences for each polymorphism were examined by chi *x*^2 ^test. Student *t*-test or Mann-Whitney test and Kruskall-Wallis test, when appropriate, were used for comparing TF and TFPI levels between two or more groups. Two-tailed probability values of 0.05 or less were considered statistically significant. Exact p-values are given, except when p < 0.001 or p > 0.2. All statistical analyses were performed in SPSS 16.0 (SPSS Inc., Chicago, IL, USA).

## Results

### Clinical characteristics

Baseline characteristics of the included CHD patients are shown in Table 1. All patients were optimally treated, thus, 100% were on aspirin and 98% on statin treatment. Blood samples for genotyping in the CHD group was detectable in 996 samples. For phenotype analysis, only performed in the CHD population, measurements of TF were complete in 983 individuals and of TFPI in 1000.

**Table 1:** Characteristics of the CHD population (n = 1001).

Age (years, mean (range))	62 (36-81)
Men/Women (%)	783/218 (78/22)
Diabetes Mellitus n (%)	200 (20)
Myocardial infarction n (%)	436 (44)
Hypertension n (%)	553 (56)
SBP (mmHg)	139.4 (19.3)
DBP (mmHg)	82.1 (9.7)
Current smokers n (%)	204 (20.4)
BMI (kg/m^2^)	27.9 (11.5)
Total cholesterol (mmol/l)	4.6 (1.0)
HDL cholesterol (mmol/l)	1.3 (0.4)
LDL cholesterol (mmol/l)	2.5 (0.8)
Triglycerides (mmol/l)	1.6 (1.1)
Fasting glucose (mmol/l)	6.0 (1.9)
HbA1c (%)	6.0 (0.9)
Medication %	
Statins	98
Aspirin	100
β-Blockers	76
Nitrates	22
ACE inhibitors	26

Values are mean (SD) or number (proportions) if not otherwise stated.

SBP: systolic blood pressure; DBP: diastolic blood pressure; BMI: body mass index; ACE:

angiotensin converting enzyme.

### Genotype distribution and allelic frequencies

The distribution of the TF and TFPI SNPs in CHD patients and healthy controls is shown in Table 2. All SNPs conformed to the Hardy-Weinberg equilibrium. The two TF polymorphisms at positions -1812 and -603 were found to be completely concordant in 1199 of the total of 1200 individuals. No significant case-control differences in frequencies of any TF polymorphisms were observed, also not when analyzing for the TF I haplotype. When analyzing the three variants of the TF -1812 and the TF -603 SNP's separately, the results were similar. Also for the TFPI SNPs no differences in frequencies between CHD and healthy controls were found.

**Table 2:** Frequency of the TF and TFPI polymorphisms in CHD patients and healthy controls

Genotype	CHD n (%)		Controls n (%)		p
TF -1812		996		204	
CC	327	(32.8)	63	(30.9)	
CT	466	(46.8)	102	(50.0)	>0.2
TT	203	(20.4)	39	(19.1)	
Allele T frequency	0.438		0.441		
					
TF -603		996		204	
AA	326	(32.7)	63	(30.9)	
AG	466	(46.8)	102	(50.0)	> 0.2
GG	204	(20.5)	39	(19.1)	
Allele G frequency	0.439		0.441		
					
TF 5466		996		204	
AA	879	(88.3)	181	(88.7)	
AG	112	(11.3)	22 )	(10.8	> 0.2
GG	5	(0,5)	1	(0.5)	
Allele G frequency	0.061		0.059		
					
TFPI-399		996		204	
CC	758	(76.1)	158	(77.5)	
CT	224	(22.5)	41	(20.1)	> 0.2
TT	14	(1.4)	5	(2.5)	
Allele T frequency	0.127		0.125		
					
TFPI -287	996			204	
TT	723	(72.6)	148	(72.6)	
TC	245	(24.6)	55	(27.0)	> 0.2
CC	28	(2.8)	1	(0.5)	
Allele C frequency	0.151		0.140		
					
TFPI -33		996		203*	
TT	512	(51.4)	102	(50.3)	
TC	405	(40.7)	87	(42.9)	> 0.2
CC	79	(7.9)	14	(6.9)	
Allele C frequency	0.283		0.283		
					
Haplotypes					
TF I	65	(6.5)	13	(6.4)	> 0.2

Calculations are performed with comparison of the heterozygous and homozygous pooled,

versus the wild type.

Haplotypes: TFI is the combination of TF -1812 CC and TF 5466 AG/GG

p-values refer to differences between CHD patients and controls.

*The TFPI -33 polymorphism was detectable in 203 out of 204 controls.

In the CHD population, the differences in genotype distribution between gender and in patients with T2DM or not, are shown in Table 3. The TF 5466 polymorphism was significantly more frequent in women, as compared to men (OR 2.23, 95% CI 1.48-3.37, p < 0.001), and this was also true when analyzing for the TF I haplotype (OR 2.56 95% CI 1.52-4.31, p < 0.001). The TF -1812 and the TF -603 polymorphisms were less, but not significantly, abundant in women (p = 0.124 and 0.115, respectively). The TFPI -399 polymorphism was more common in women (p = 0.075). No difference in frequencies was seen for any genotype between patients presenting with T2DM or not, or between patients who had previously experienced MI or not (data not shown).

**Table 3:** Association between TF and TFPI polymorphisms in subgroups of the CHD population

Genotype	No with T2DM+/-	% with T2DM	p-value	No of Male/Female	% Female	p-value
TF -1812						
CC	71/256	22		246/81	25	
CT	93/373	20	>0.2	366/100	21	0.124
TT	35/168	17		166/37	18	
TF -603						
AA	71/255	22		245/81	25	
AG	93/373	20	>0.2	366/100	21	0.115
GG	35/169	17		167/37	18	
TF 5466						
AA	179/700	20		703/176	20	
AG	19/93	17	>0.2	72/40	36	**<0.001**
GG	1/4	20		3/2	40	
TFPI -399						
CC	151/607	20		602/156	21	
CT	45/179	20	>0.2	166/58	26	0.075
TT	3/11	21		10/4	29	
TFPI -287						
TT	145/578	20		564/159	22	
TC	49/196	20	>0.2	194/51	21	>0.2
CC	5/23	18		20/8	29	
TFPI -33						
TT	97/415	19		399/113	22	
TC	87/318	22	>0.2	315/90	22	>0.2
CC	15/64	19		64/15	19	
Haplotypes						
TF I	13/35	20	>0.2	39/26	40	**<0.001**

Calculations are performed with comparison of the heterozygous and homozygous pooled, versus the wild type.

Haplotypes: TFI is the combination of TF -1812 CC and TF 5466 AG/GG

p-values refer to differences between disease states and gender, as related to genotype.

Due to gender differences, we evaluated the T2DM genotype distribution in the subgroup of women (n = 218) (Table 4). The TF -1812 and the TF -603 polymorphisms were significantly less frequent in CHD women with T2DM as compared to women without (OR 0.45, 95% CI 0.14-0.88, p = 0.018 for both). These associations were not observed in men (data not shown).

**Table 4:** Frequencies of TF and TFPI polymorphisms in women with CHD according to T2DM or not

Genotype	T2DM+ (46) n(%)	T2DM- (172) n(%)	p
TF -1812			
CC	24 (52.2)	57 (33.1)	**0.018**
CT	17 (37.0)	83 (48.3)	
TT	5 (10.9)	32 (18.6)	
Allele T frequency	0.294	0.428	
TF -603			
AA	24 (52.2)	57 (33.1)	**0.018**
AG	17 (37.0)	83 (48.3)	
GG	5 (10.9)	32 (18.6)	
Allele G frequency	0.294	0.428	
TF 5466			
AA	36 (78.3)	140 (81.4)	>0.2
AG	9 (19.6)	31 (18.0)	
GG	1 (2.2)	1 (0.6)	
Allele G frequency	0.120	0.096	
TFPI -399			
CC	37 (80.4)	119 (69.2)	0.133
CT	9 (19.6)	49 (28.5)	
TT	0	4 (2.3)	
Allele T frequency	0.098	0.166	
TFPI -287			
TT	34 (73.9)	125 (72.7)	>0.2
TC	9 (19.6)	42 (24.4)	
CC	3 (6.5)	5 (2.9)	
Allele C frequency	0.163	0.151	
TFPI -33			
TT	24 (52.2)	89 (51.7)	>0.2
TC	21 (45.7)	69 (40.1)	
CC	1 (2.2)	14 (8.1)	
Allele C frequency	0.251	0.282	

Calculations are performed with comparison for the heterozygous and homozygous pooled, versus the wild type.

p-values refer to differences between diabetes versus non-diabetes.

### Plasma levels of TF and TFPI according to genotypes and disease state and gender

To evaluate the influence of the investigated SNPs on the TF and TFPI plasma levels, the biomarkers were measured in the total CHD population (Table 5). Patients presenting with the TF -1812 and TF -603 polymorphisms showed significant differences in the TF levels, with the heterozygous (n = 466) having the lowest levels (p = 0.013 and 0.019, respectively). The TFPI -399 and the TFPI -33 polymorphisms were associated with significantly lower and higher TFPI total antigen levels, respectively (p < 0.001 for both).

**Table 5:** Plasma TF and TFPI levels according to genotypes in the total CHD population

Genotypes	TF pg/ml*	p		
TF -1812				
CC	150 (107,210)			
CT	138 (96,187)	**0.013†**		
TT	153 (109,203)			
TF -603				
AA	149 (107,210)			
AG	139 (97,187)	**0.019†**		
GG	153 (109,203)			
TF 5466				
AA	143 (104, 197)			
AG/GG^||^	154 (104, 197)	>0.2		
				
	Free TFPI ng/ml‡		Total TFPI ng/ml‡	p
TFPI -399				
CC	15.5 (4.6)		68.6 (14.1)	
CT/TT^||^	15.1 (5.8)	> 0.2	64.2 (14.9)	**<0.001§**
TFPI -287				
TT	15.2 (4.9)		67.2 (14.7)	
TC/CC^||^	15.7 (4.9)	0.178	68.6 (13.6)	0.183
TFPI -33				
TT	15.5 (5.2)		63.3 (13.3)	
TC/CC^||^	15.3 (4.6)	> 0.2	72.1 (14.1)	**<0.001§**

* Values are median levels (25 and 75 percentiles)

† Kruskall-Wallis test

‡ Values are mean levels (SD)

**§**Independent sample *t*-test

|| Heterozygous and homozygous are combined, due to low number of minor allele homozygous.

p-values refer to differences between genotypes.

No differences according to the presence of T2DM or gender were observed (Additional file 1). The patients who had previously experienced MI (n = 436) had significantly higher TFPI total antigen levels as compared to the subjects without MI (p = 0.008).

**Figure 1:**
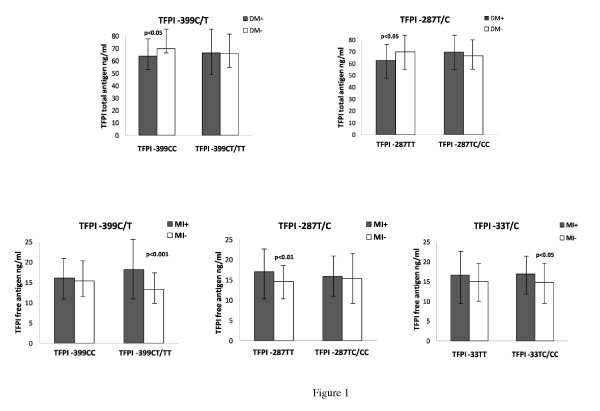
**Plasma levels of TFPI in women according to genotypes, T2DM and MI**. Levels of TFPI total antigen according to genotypes and T2DM (n = 46) in upper panel. Levels of TFPI free antigen according to genotypes and MI (n = 71) in lower panel. Values are mean, with error bars representing SD.

Based on the gender differences in genotypes, we further analyzed for phenotype in women alone according to disease state (Additional file 2). TFPI total antigen levels were lower in females with T2DM (n = 46) as compared to those without (p = 0.093). When analyzing specifically for the different genotypes, the TFPI levels in T2DM women bearing the major allele of TFPI -399 and TFPI -287 were significantly lower compared to women without T2DM (p < 0.05 for both) (Figure 1). TFPI free antigen levels were significantly higher in females previously suffering MI (n = 71) as compared to non-MI women (p = 0.013) (Additional file 2), mainly observed in those bearing the minor allele of the TFPI -399 and the TFPI -33 polymorphisms, and in subjects homozygous for the major TFPI -287 allele (Figure 1). In men, the TFPI total antigen was significantly higher in MI patients, irrespective of genotype (p = 0.025) (data not shown).

## Discussion

In this study we could show that the investigated genetic variants of the TF and the TFPI genes were not differently distributed between CHD patients and healthy controls. It seems therefore that these polymorphisms alone not necessarily dispose for the disease. They might, nevertheless, contribute to an increased susceptibility. As CHD and arterial thrombosis are multifactorial disorders, including both genetic and environmental factors, we explored further if these genetic variants were linked to gender, T2DM and subtypes of CHD (MI).

### Frequencies in the CHD population and controls

The observed frequencies of the SNPs in the CHD population and healthy controls are mainly in line with other reports [20-26], also investigating CHD in Caucasians. In a Swedish study, in patients with acute coronary syndrome, the TF 5466A/G polymorphism was associated with cardiovascular death, and the CG haplotype, by TF -1812C/T and TF 5466 A/G, was associated with a 3-fold increased risk of death [22]. In our study no association between any of the investigated polymorphisms and the presence of stable CHD was observed. It should be noted that the unbalanced number of cases and controls might have influenced the results and the control group was also seven years younger.

We could also not find any association in the subgroup of CHD patients with previous MI. As for the TF-603 A/G polymorphism and the association to cardiovascular events, diverging results are reported [20,21,23]. No case-control differences in frequencies with regard to CHD have been observed for the TFPI polymorphisms [24,25].

### Genotypes and phenotypes in the total CHD population

In our CHD population the heterozygotes for the two linked TF polymorphisms, the TF-1812C/T and the TF-603A/G, presented with significantly lower TF plasma levels as compared to the two homozygous genotypes, pointing to a potential protecting role of these polymorphisms. This is somewhat in contrast to the findings from smaller sub-sets in the ECTIM and PATHROS studies [20]. These results were, however, obtained in healthy individuals. We observed no genotype-phenotype association for the TF 5466 SNP, which is in line with the main results from Mälarstig et al. [22]. They could, however, show that the TF mRNA expression was influenced by the SNP. In our study, the TFPI -399C/T and the TFPI -33 T/C polymorphisms were associated with lower and higher TFPI total antigen levels, respectively, in line with other reports [25,26]. Thus, both protective and harmful role of the single nucleotide polymorphisms might be present. The TFPI -287T/C did not affect the TFPI plasma levels significantly.

### Genotypes and phenotypes as related to gender

The higher prevalence of the TF 5466A/G polymorphism in women with CHD has previously not been reported neither the tendency towards a higher prevalence in women for the TFPI -399C/T polymorphism. As the TFPI -399C/T SNP seems to lead to lower levels of TFPI, as shown in our study, and the TF 5466A/G SNP seems to give higher mRNA levels, shown in another study [22], a possible prothrombotic state with a combined genotype in women with CHD might be suggested.

### Genotypes and phenotypes in women with T2DM

The T and G allele of the TF -1812C/T and the TF -603A/G polymorphisms, respectively, were significantly more frequent in women without T2DM as compared to women with diabetes. As the heterozygous state was associated with significantly lower TF levels, women with CHD not presenting with diabetes may be less susceptible to thrombotic events, and a possible causal role of these genetic polymorphisms in the pathogenesis of atherothrombosis in T2DM may be considered. Patients presenting with diabetes have been shown to be more hypercoagulable and prone to acute coronary events, partly through the activation of the TF pathway [28]. As hyperglycemia and hyperinsulinemia is common in patients with T2DM, we propose that the recently shown contribution to the procoagulant state [18], may partly be TF genotype dependent.

The observed lower TFPI total antigen in the present study in women with diabetes was only significant for the TFPI -399 CC and the TFPI -287 TT genotypes, which my indicate that the regulation of TFPI by hyperglycemia to some degree is related to the TFPI genotype. However it should be emphasized that the less frequent genotypes also may play a role.

### Genotypes and phenotypes in women with MI

The frequencies for all SNPs were similar for women with and without previous MI and plasma levels of TF did not differ between the groups. Higher TFPI free antigen levels were observed for this group, especially in individuals bearing the TFPI -399 CT/TT, the TFPI -287 TT and the TFPI -33TC/CC genotypes. This association was not observed in men (data not shown). The importance of TFPI in atherothrombotic disease is still controversial. Higher plasma levels of TFPI, which have been reported in CHD patients [9], may reflect a compensatory mechanism due to activation of the TF coagulation pathway, but may also reflect endothelial dysfunction in the atherosclerotic process. The free pool of TFPI includes the full-length TFPI and generally reflects the changes in endothelial cell-associated TFPI [29], whereas the free form with its strong anticoagulant activity is thought to be important for the function of TFPI *in vivo *[30]. It may thus be suggested that women are more prone to develop a dysfunctional endothelium. Whether this gender discrepancy in plasma levels of TFPI may be important for the disease progression remains to be elucidated and require further investigation. Notable, as almost all patients were on statin treatment no corrections for cholesterol, of possible importance for the measurements of TFPI total antigen, have been performed.

## Conclusion

Genetic variations of the TF and TFPI genes seem to be associated with gender in the present population of CHD patients. Women presenting with T2DM showed a different pattern in TF and TFPI genotypes, as compared to women without diabetes, partly related to their respective phenotypes. The phenotype differences observed are minor and within the normal range. However, recent studies have shown that minor increase and decrease in prothrombotic factors and inhibitors, respectively, may induce thrombin generation and subsequent thrombus formation. Thus, a possible enlargement of a TF/TFPI ratio due to genetic variants, may contribute to a hypercoagulable state in CHD, which may be more important for clinical events in women with diabetes. It should, however, be underlined that these results have to be confirmed in studies more specifically designed, due to low number in the different subgroups.

## Conflict of interests

The authors declare that they have no competing interests.

## Authors' contributions

TBO conducted the study and was responsible for all analysis, drafted and revised the manuscript. AÅP contributed to the study protocol, acquired data and discussed the manuscript. TW contributed in the interpretation of results and discussed the manuscript. HA contributed to the study protocol, the interpretation of results and discussed the manuscript. IS conducted the study, contributed to the interpretation of results, drafted and revised the manuscript, and gave the final approval of the version to be published. All authors read and approved the final manuscript.

## Authors' information

TBO: MSc, PhD student

AÅP: MD, PhD student

TW: MD, PhD student

HA: Professor MD PhD

IS: Professor PhD

## Supplementary Material

Additional file 1 Plasma TF and TFPI levels according to T2DM (DM), MI and gender in the total CHD population (n = 1001) Click here for file

Additional file 2 Plasma TF and TFPI levels in women according to T2DM (DM) and MI Click here for file
