# Supplementary material for: Gender differences of polymorphisms in the TF and TFPI genes, as related to phenotypes in patients with coronary heart disease and type-2 diabetes
Source: Thromb J. 2010 May 5;8:7. doi: 10.1186/1477-9560-8-7 (PMC2882354; doi:10.1186/1477-9560-8-7)
Supplement: Additional file 1 — Plasma TF and TFPI levels according to T2DM (DM), MI and gender in the total CHD population (n = 1001) [file 1477-9560-8-7-S1.DOC]

Additional file 1 Plasma TF and TFPI levels according to T2DM (DM), MI and gender in the total CHD population (n=1001)

| Disease | n | TF pg/ml* | p | Free TFPI ng/ml† | p | Total TFPI ng/ml† | p |
| --- | --- | --- | --- | --- | --- | --- | --- |
| DM +  - | 200  801 | 143 (97,190)  143 (104,200) | >0.2 | 15.2 (4.0)  15.4 (5.1) | >0.2 | 66.7 (14.3)  67.8 (14.4) | >0.2 |
| MI +  - | 436  565 | 145 (106,199)  143 (102,194) | >0.2 | 15.6 (4.9)  15.2 (4.9) | >0.2 | 69.0 (14.2)  66.5 (14.5) | **0.008** |
| Male  Female | 783  218 | 143 (105,194)  148 (98,209) | >0.2 | 15.3 (4.8)  15.5 (5.3) | >0.2 | 67.6 (14.2)  67.7 (15.0) | >0.2 |

* Values are median (25 and 75 percentiles)

† Values are mean (SD)

p-values refer to differences between disease states and gender
