# Supplementary material for: Gender differences of polymorphisms in the TF and TFPI genes, as related to phenotypes in patients with coronary heart disease and type-2 diabetes
Source: Thromb J. 2010 May 5;8:7. doi: 10.1186/1477-9560-8-7 (PMC2882354; doi:10.1186/1477-9560-8-7)
Supplement: Additional file 2 — Plasma TF and TFPI levels in women according to T2DM (DM) and MI [file 1477-9560-8-7-S2.DOC]

Additional file 2 Plasma TF and TFPI levels in women according to T2DM (DM) and MI

| Disease | n | Sol TF pg/ml* | p | Free TFPI ng/ml† | p | Total TFPI ng/ml† | p |
| --- | --- | --- | --- | --- | --- | --- | --- |
| DM +  - | 46  172 | 143 (91,181)  150 (102,213) | >0.2 | 15.2 (3.4)  15.5 (5.8) | >0.2 | 64.4 (14.2)  68.6 (15.1) | 0.093 |
| MI +  - | 71  147 | 140 (106,212)  150 (97,203) | >0.2 | 16.8 (6.1)  14.8 (4.8) | **0.013** | 70.0 (15.1)  66.6 (14.8) | 0.122 |

* Values are median (25 and 75 percentiles)

† Values are mean (SD)

p-values refer to differences between disease states
